# Platelet, Plasma, Urinary Tryptophan-Serotonin-Kynurenine Axis Markers in Hyperacute Brain Ischemia Patients: A Prospective Study

**DOI:** 10.3389/fneur.2021.782317

**Published:** 2022-01-11

**Authors:** Luigi F. Saccaro, Fernando Pico, Marie-Laure Chadenat, Olivier Richard, Jean-Marie Launay, Brigitte Bastenaire, Philippe Jullien, Jerôme Lambert, Vincent Feuga, Maryline Macquet, Jacques Callebert, Yves Lambert, Odile Spreux-Varoquaux

**Affiliations:** ^1^Neurology and Stroke Care Unit, Versailles Hospital, Le Chesnay, France; ^2^Department of Emergency, Versailles Hospital, Le Chesnay, France; ^3^INSERM U942, Hôpital Lariboisière, Paris, France; ^4^Department of Hematology, Versailles Hospital, Le Chesnay, France; ^5^Department of Anesthesia-Intensive Care, Versailles Hospital, Le Chesnay, France; ^6^Saint-Louis Hospital, Department of Biostatistics and Medical Information, University of Paris, Paris, France; ^7^Department of Psychiatry , Versailles Hospital, Le Chesnay, France; ^8^Pharmacology, Service de Biologie Médicale, Versailles Hospital, Le Chesnay and University of Versailles, Saint-Quentin en Yvelines, France

**Keywords:** serotonin, tryptophan, kynurenine, acute ischemic stroke (AIS), transient ischemic attack (TIA), biomarkers, 5-HT

## Abstract

**Background and Purpose:** Ischemic stroke is one of the most common causes of morbidity and mortality and has numerous clinical mimics. Previous studies have suggested a potential role of the tryptophan-serotonin (5-HT)-kynurenine (TSK) axis in ischemic stroke. Studies assessing this axis in the hyperacute phase of ischemic stroke (<4.5 h) are lacking. This prospective study thus evaluates the TSK axis in transient ischemic attack (TIA) and hyperacute ischemic stroke (AIS) patients.

**Methods:** This study included 28 patients (24 AIS and 4 TIA) and 29 controls. The blood and urine samples of patient were collected within 4.5 h of symptoms onset (day 0, D0), then at 24 h and 3 months. Control blood and urine samples were collected once (D0). The TSK axis markers measured were platelet serotonin transporter (SERT) and 5-HT_2A_ receptor (5-HT_2A_R) densities and platelet, plasma, and urinary 5-HT, plasma and urinary 5-hydroxyindole acetic acid (5-HIAA), and plasma kynurenine and tryptophan (TRP) levels.

**Results:** At D0, patients exhibited a lower (*p* = 10^−5^) platelet SERT density, higher (*p* < 10^−6^) platelet 5-HT_2A_R density, higher (*p* = 10^−5^) plasma kynurenine/tryptophan (K/T) ratio, and higher urinary 5-HT (*p* = 0.011) and 5-HIAA (*p* = 0.003) levels than controls.

**Conclusions:** We observed, for the first time, a hyperacute dysregulation of the serotonergic axis, and hyperacute and long-lasting activation of the tryptophan-kynurenine pathway in brain ischemia.

## Graphical Abstract

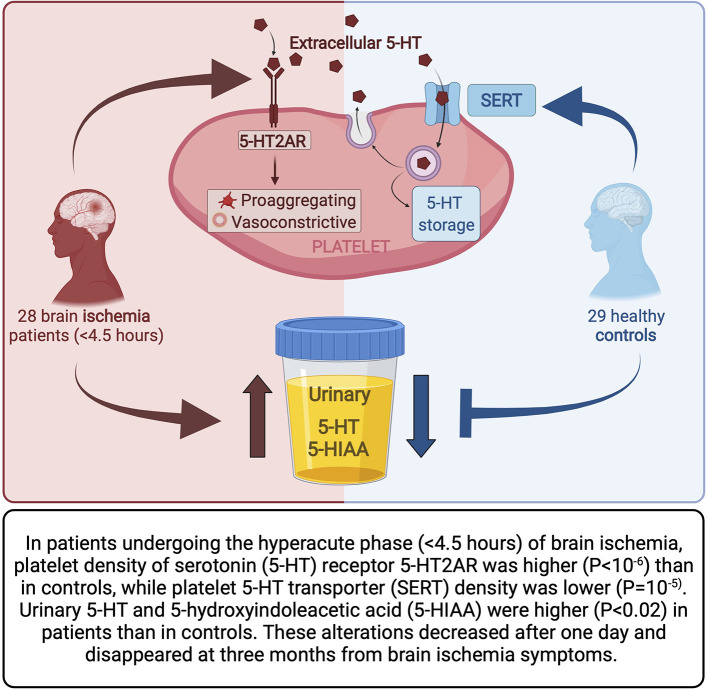

## Introduction

Stroke is the second most common cause of disability-adjusted life-years worldwide, affecting 15 million people annually (1). Approximately 85% of strokes are ischemic but we still lack biomarkers that can help differentiate brain ischemia from its many mimics (2). Pivotal players in this process are endothelial dysfunction and platelet aggregation. Although activation of the tryptophan (TRP)-kynurenine axis (the main route of TRP catabolism) may play a role in ischemic stroke, a recent systematic review highlighted the absence of clinical studies on the topic (3). TRP-kynurenine activation in acute ischemic stroke may possibly be a consequence of increased activity of the initiating enzyme of this pathway (i.e., indoleamine 2,3-dioxygenase, IDO), which is upregulated by inflammatory stimuli (3–5). TRP is internalized by small intestine enterochromaffin cells, which then hydroxylate and decarboxylate it to 5-HT. 5-HT is released into the bloodstream and stored in vesicles in platelets through a serotonin transporter (SERT), and, when released from platelets, mainly catabolized into inactive 5-hydroxyindole acetic acid (5-HIAA) *via* the monoamine oxidase A (MAO-A), contained in various tissues, such as liver, adipocytes, and vascular endothelium. Certain 5-HT receptors, such as the 5-HT_2A_R present on platelet membranes, have platelet pro-aggregating and vasoconstrictive effects (6). 5-HT and pro-serotonergic drugs, such as selective serotonin reuptake inhibitors (SSRIs), have platelet pro-aggregating actions and vasoconstrictive effects, both may play a role in hyperacute ischemic stroke (AIS) (7). Indeed, SERT polymorphisms have been shown to be associated with a higher ischemic stroke risk (8). Nevertheless, only a few previous studies have explored certain markers of the tryptophan-serotonin (5-HT)-kynurenine (TSK) axis 24 h after brain ischemia (3, 5), and there are no prospective studies that have measured TSK axis markers in blood and urine in the hyperacute phase (<4.5 h) of brain ischemia.

We thus aimed at evaluating TSK axis markers in blood and urine, such as, for the first time, platelet SERT and 5-HT_2A_R, during the hyperacute phase (<4.5 h) of brain ischemia, as well as with repeated measurements at day 1 (D1) and 3 months (M3).

## Methods

Briefly, blood and urine samples of patients with acute brain ischemia were collected within 4.5 h of symptoms onset (day 0, D0) and then at 24 h (D1) and M3 from symptoms onset. The blood and urine samples of controls were collected once (D0). TSK axis markers were measured and compared between patients and controls and between different time points, as detailed in the following paragraphs. Detailed inclusion and exclusion criteria, clinical evaluation and demographic data of the participants, and a flowchart of the study design are provided in the Supplementary Material.

### Study Population—Recruitment

In this prospective, monocentric, observational study, 53 consecutive adults (>18 years of age) patients with suspected AIS and candidates for intravenous thrombolysis were included from September 10, 2012 to November 27, 2014 at the Stroke center of Versailles Hospital (France), as detailed in Supplementary Figure 1. After screening of 53 patients, 12 patients were excluded due to an alternative diagnosis and negative MRI, 13 due to informed consent problems. In total 28 patients were included in this study, 24 diagnosed with ischemic stroke and 4 with transient ischemic attack (TIA). The hospital was pre-notified of all patients and they underwent brain imaging upon hospital arrival <4.5 h from symptoms onset. In addition, 60 controls without clinical evidence of cerebral ischemia were screened and 29 enrolled, including healthy volunteers or historical controls, i.e., inpatients that had been examined before orthopedic surgery for other clinical studies. Further details are provided in the Supplementary Material.

### Clinical Evaluation of Patients

Patients were clinically evaluated in the Emergency Department or Stroke Unit of the Versailles Hospital, where a detailed clinical history was taken, and further examinations were performed. Further details are provided in the Supplementary Material and in Supplementary Table 2.

### Statistical Analyses

Non-identifying data from clinical and biological measurements were analyzed using Statistical Package for the Social Sciences (SPSS) and Statistical Analysis System (SAS) software. The Shapiro–Wilk normality test was used to evaluate the data distribution. Student's *T*-test was used for comparisons between patients and controls for normally distributed data and Pearson's correlation coefficient was used to evaluate relationships between variables. Qualitative data were compared using Fisher's exact test and quantitative non-normally distributed data using Wilcoxon's non-parametric test for paired samples when appropriate. Spearman's non-parametric correlation coefficient was used to evaluate relationships between non-normally distributed variables. All tests were two-sided and a *p* < 0.05 was considered significant. Age and tobacco exposure (to quantify smoking and exposure to environmental tobacco smoke) were included as co-variables when making comparisons. Drug therapies that could interfere with the biological parameters being evaluated, especially serotonin, were recorded. In particular, subjects were divided into subgroups based on SSRI usage at the time of inclusion.

### Biological Sampling and Parameters

All controls and patients were studied in the Versailles Hospital and the same biological parameters were measured using the same techniques and equipment (9–11). The blood and urine samples at 3 months (M3) from symptoms onset were collected during the neurological follow-up consultation.

The TSK axis markers measured were platelet SERT and 5-HT_2A_R densities and platelet, plasma, and urinary 5-HT, plasma and urinary 5-HIAA, plasma kynurenine, and total (free + bound) tryptophan (TRP) levels. Besides, platelet aggregation and plasma homocysteine levels were measured. Sample collection and biochemical determinations were performed according to the procedures previously described by our group (9–11) for plasma TRP and kynurenine, plasma and platelet 5-HT, and plasma 5-HIAA using various standardized specific and sensitive high-performance liquid chromatography (HPLC) with coulometric detection methods, except for platelet 5-HT, for which blood was used instead of serum. The urinary 5-HT and 5-HIAA determinations used were derived from plasma 5-HT and 5-HIAA methods (10, 11). The results of the urinary 5-HT and 5-HIAA assays were adjusted for renal function. The molar ratio between urinary 5-HIAA and 5-HT levels was calculated and used as an indirect index of monoamine oxidase-A (MAO-A, the enzyme catabolizing 5-HT to 5-HIAA) activity, and the molar ratio between plasma kynurenine and total TRP levels x100 was calculated and used as an indirect index of indole amine 2,3-dioxygenase (IDO) activity. Platelet SERT and 5-HT_2A_R densities were determined through radioligand bindings. They were carried out in duplicate tubes containing 50 mM Tris buffer (pH 7.7), [^125^I]DOI 2.5 nM (5-HT_2A_R) or [^3^H]paroxetine 1 nM (SERT), and 40 μl of platelet membrane suspension with (nonspecific bindings) or without (total bindings) 1 μM ketanserin (5-HT_2A_R) or 10 μM fluoxetine (SERT) in a total volume of 100 μl. After incubation for 2 h at 37°C, filtration (GF/B filters) and washing (3 × 5.0 ml cold buffer + 0.01% BSA) filters were counted.

To measure platelet aggregation, 0.5 μM and 2 μM adenosine diphosphate (ADP), 0.5 μM ADP + 10 μM 5-HT, and 2 μM ADP + 10 μM 5-HT were used as aggregate inducers (11).

The blood and urine collection of patients and controls were carried out at the same times and in the same manner, minimizing any potential bias from these sources on the results.

## Results

### Results of Platelet Analyses

**Figure 1:**
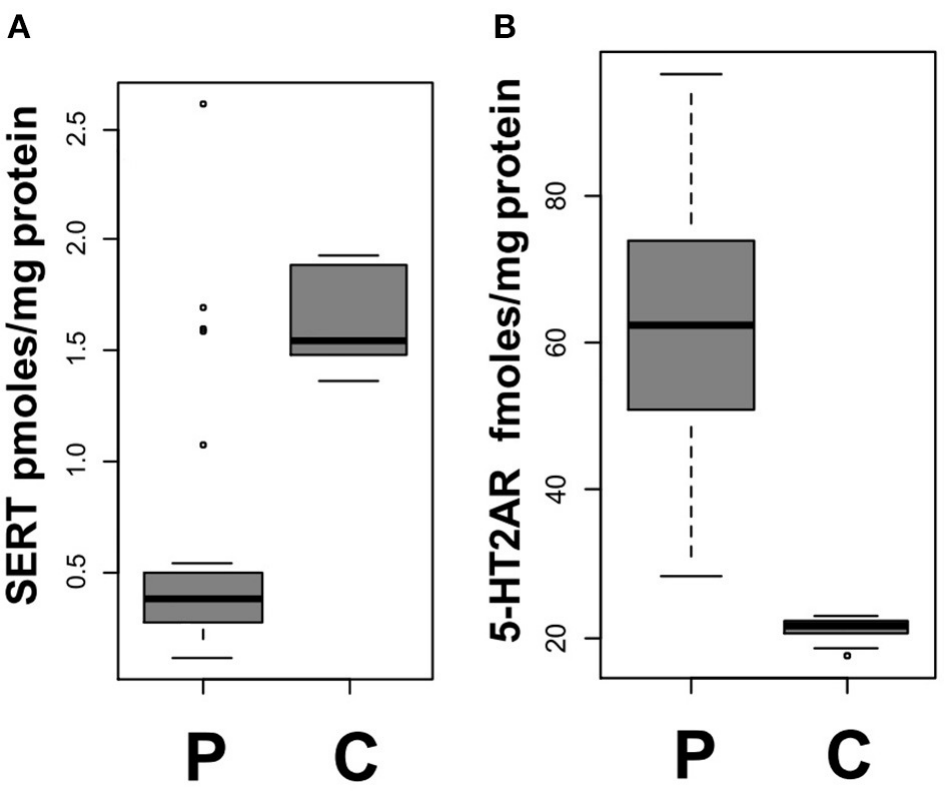
Platelet markers at day 0. **(A)** Blood platelet 5-HT transporter (SERT) density in patients (P) and controls (C) (*p* = 10^−5^). **(B)** Blood platelet 5-HT receptor (5-HT2AR) density in patients (P) and controls (C) (*p* < 10^−6^). Each gray square indicates the interquartile range (IQR) and the black line the median.

At D0, the platelet SERT density was lower (*p* = 10^−5^) in patients [median: 0.38 (interquartile range, IQR: 0.27–0.5) pmol/mg protein] than controls [1.55 (1.48–1.88)] (Figure 1A). The platelet 5-HT_2A_R density was higher (*p* < 10^−6^) in patients [62.5 (51.15–71.05) fmol/mg protein] than controls [21.5 (20.5–22.3)] (Figure 1B).

*In vitro* addition of 5-HT 10 μM to ADP to platelets samples, even at minimal concentration of ADP (0.5 μM), significantly increased platelet aggregation intensity and speed for both patients and controls (*p* < 10^−5^), with no significant differences in platelet aggregability between the two groups (Supplementary Tables 7, 8).

There were no significant differences in these parameters (i.e., platelet SERT and 5-HT_2A_R densities, plasma, urinary, and platelet 5-HT) between male (*n* = 14) and female (*n* = 14) patients, as detailed in Supplementary Table 4, nor between participants taking SSRI and those who were not (*p* > 0.05 for each comparison). Further details are provided in the Supplementary Material.

There was no significant difference between patients and controls or between patients at D0, D1, or M3 for platelet 5-HT.

Further details are provided in the Supplementary Material and Supplementary Table 3.

### Results of Urinary Analyses

**Figure 2:**
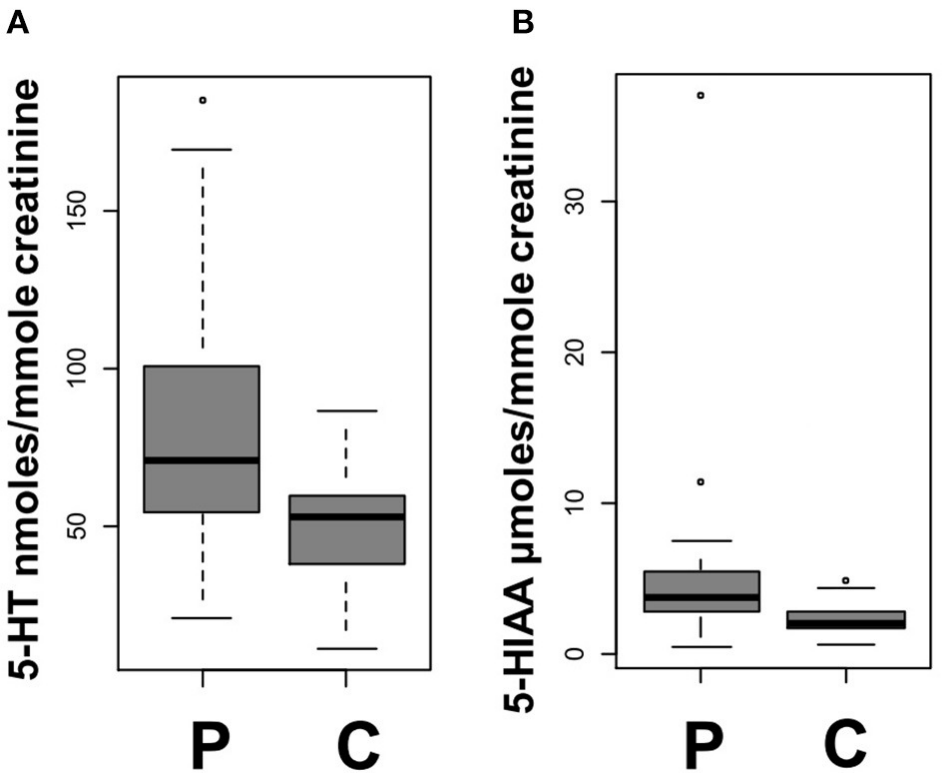
Urinary markers at day 0. **(A)** Urinary 5-HT in patients (P) and controls (C) (*p* = 0.011). **(B)** Urinary 5-HIAA in patients (P) and controls (C) (*p* = 0.003). Each gray square indicates the IQR and the black line the median.

At D0, urinary 5-HT levels were higher (*p* = 0.011) in patients [71.05 (54.08–100.48)] than controls [52.9 (37.55–59.15) nmol/mmol creatinine] (Figure 2A). Urinary 5-HIAA levels were also higher (*p* = 0.003) in patients [3.8 (2.9–5.6) μmol/mmol creatinine] than controls [2.09 (1.7–2.86)] (Figure 2B) at D0.

The D0 urinary 5-HT levels of patients were significantly higher than those at D1 (*p* = 0.016) and M3 (*p* = 0.007), whereas there was no difference between the D1 and M3 urinary 5-HT values (*p* = 0.461).

There was no significant difference in D0 MAO-A activity index between patients and controls, nor between D0, D1, or M3 for the other measured urinary parameters in patients (urinary 5-HIAA and MAO-A activity index), as detailed in Supplementary Figure 3. There was no significant difference in urinary 5-HT and urinary 5-HIAA between male and female subjects (Supplementary Table 4).

Further details are provided in the Supplementary Material and Supplementary Table 3.

### Results of Plasma Analyses

Plasma kynurenine/tryptophan (K/T) ratio (IDO activity index), was higher (*p* = 10^−5^) in patients [5.56 (4.72–6.67)] than controls [3.74 (2.86–4.52)] at D0. The plasma K/T ratio of patients at both D0 and D1 was lower than at M3 (*p* < 0.05).

There were no significant differences in these and others plasma parameters (i.e., plasma 5-HT, plasma 5-HIAA, and plasma K/T ratio) between male (*n* = 14) and female (*n* = 14) patients, as detailed in Supplementary Table 4, nor between participants taking SSRI and those who were not (*p* > 0.05 for each comparison).

There was no significant difference between patients and controls or among patients at D0, D1, or M3 for the other measured blood biological parameters, i.e., plasma 5-HT, plasma 5-HIAA, and plasma homocysteine. There was no significant difference between patients and controls or between patients at D0, D1, or M3 for the other measured plasma biological parameters.

In addition, we found no significant correlation between plasma homocysteine levels and those of other D0 TSK markers.

Further details are provided in the Supplementary Material and Supplementary Table 3.

### Sub-Analyses

We performed a sub-analysis excluding patients with TIA (*n* = 4). At D0, platelet SERT density remained significantly lower (*p* < 10^−5^) in patients than controls. Platelet 5-HT_2A_R density remained significantly higher (*p* < 10^−6^) in patients than controls, as were urinary 5-HT (*p* = 0.03) and urinary 5-HIAA (*p* = 0.008). The D0 plasma K/T ratio of patients remained higher than controls (*p* < 10^−4^). The plasma K/T ratio of patients at D0 and D1 remained lower than at M3 (*p* < 0.05 for all comparisons; Supplementary Material and Supplementary Table 5).

Differences in blood markers (i.e., platelet SERT density, platelet 5-HT_2A_R density, and plasma K/T ratio) and urinary markers (i.e., urinary 5-HT and urinary 5-HIAA) remained significant (*p* < 0.0001 and *p* < 0.022, respectively) after excluding the six patients taking SSRI from the analyses.

Globally, patients were slightly older than controls (*p* = 0.0483). Further details are provided in the Supplementary Material.

### Non-Significant Results

There were no significant differences between patients and controls for sex or tobacco exposure (Supplementary Table 1), two important confounders for the interpretation of serotonergic parameters data. However, it ought to be noted that the study was not powered enough for detecting sex differences in these parameters. Among all participants, four patients (three women and one man) were tobacco smokers, and six female patients were taking SSRI. There was no significant correlation between any of the measured TSK axis markers and the NIHSS score of patients at the emergency department or in the stroke unit of hospital.

Further details are provided in the Supplementary Material and Supplementary Table 3.

## Discussion

Overall, as recapitulated in the graphical abstract, this study provides the first evidence of abnormalities of the TSK axis (**i**) for patients in the hyperacute phase (<4.5 h) of cerebral infarction relative to controls and (**ii**) for intra-subject measurements obtained from patients after 1 or 90 days from symptoms onset.

The 5-HT levels are mainly regulated by SERT and MAO-A. The marked decrease in platelet SERT and increase in platelet 5-HT_2A_R densities observed in patients may be possible mechanisms that increase the ischemic risk or reflect the presence of a thrombotic process, as such alterations could promote platelet aggregation, local platelet release of 5HT, and local pro-ischemic vasoconstriction. Although, this is the first time that this clinical finding has been reported, it is in accordance with results from a recent study on 834 patients with AIS and TIA showing that genetic polymorphisms associated with increased SERT expression are linked to a lower risk of cerebral ischemia (12). Additionally, it is consistent with the results of studies showing a decrease in platelet SERT densities in drug-free depressed patients, further reduced by SSRIs (11), and an increased AIS risk associated with SSRI treatment (8, 12). Furthermore, an increase in platelet 5-HT_2A_R density and aggregation response has been observed with certain antidepressant treatments, e.g., clomipramine (11). The increased *ex vivo* platelet aggregability found both in patients and controls after the addition of 5-HT confirm the role of this neurotransmitter in platelet aggregation. On the other hand, the absence of difference between patients and controls in this test *ex vivo* cannot be interpreted as a reflection of *in vivo* processes.

The fact that alterations were found in the levels of urinary serotonergic markers but not in plasma is consistent with the very short half-life of 5-HT in plasma. Furthermore, patients showed increased urinary 5-HT levels only in the first hours following brain ischemia, further supporting the hypothesis of a transient serotonergic storm in the setting of brain ischemia.

The higher plasma K/T ratio (IDO activity index) observed in patients with TIA and AIS is in accordance with the results of previous AIS studies, that reported lower TRP levels and higher kynurenine levels in patients with AIS than in controls (4, 5, 13). At biochemical level, kynurenine and serotonin pathways are linked by their common precursor tryptophan, which influences both serotonergic and kynurenine pathways. We found the TRP-kynurenine pathway to be already activated in the hyperacute phase (<4.5 h) of brain ischemia, not just within 24 h of symptoms onset, as previously reported (13, 14), and it was still activated 3 months later. The K/T ratio has been found to correlate with brain infarction volumes in patients with stroke (14). Although the exact effects of kynurenine and its catabolites are still debated (15), as mentioned in the introduction, the TRP-kynurenine pathway appears to be activated in acute ischemic stroke, possibly as a consequence of increased activity of the initiating enzyme of this pathway, i.e., indoleamine 2,3-dioxygenase (IDO), which is upregulated by inflammatory stimuli (3–5). This pathway generates neurotoxic and pro-apoptotic catabolites (3).

This study thus further confirms the activation of the TRP-kynurenine catabolic pathway in acute brain ischemia and shows that such an alteration is already present a few hours after symptoms onset and is long-lasting.

Such findings may have important clinical implications in the quest for biomarkers specific for the hyperacute phase of brain ischemia [differentiating it from its numerous mimics (2)].

The present findings are of interest also considering that pro-serotonergic, anti-depressant drugs, such as SSRI, have platelet pro-aggregating actions and vasoconstrictive effects, and may play a role in AIS. Thus, these results add to previous knowledge on the relationship between stroke and depression, with the dysregulation of the serotonergic system being one link between these two conditions.

However, both technological advancements and further studies are needed to translate the above results to real-life prehospital evaluation of patients with suspected AIS or TIA.

## Limitations

This study has some limitations. Due to its preliminary nature, the sample size is small, in particular, the numerosity of patients with TIA is low. Thus, the study is underpowered to allow comparisons between patients with TIA and AIS, who were analyzed together, as they all suffered a cerebral ischemic insult. While exclusion criteria were different between controls and patients, patients were not taking other antidepressants apart from SSRI, nor monoamine oxidase inhibitors (MAOIs), opioids, triptans, and valproate. Only one patient was treated with L-dopa. The therapies of patients included antihypertensive drugs, anticoagulants, antiplatelets, statins, bronchodilators, antidiabetics, and antiulcer drugs. We did not collect information on herbal medicine. Furthermore, patients were slightly older than controls, but this weakly significant difference could hardly explain, alone, the very significant difference we observed in serotoninergic parameters. Control patients did not undergo MRI to exclude silent ischemic strokes but the low likelihood of such events and the differences observed between the groups suggest that this limitation did not compromise the soundness of the study.

## Data Availability Statement

The raw data supporting the conclusions of this article will be made available by the authors, without undue reservation.

## Ethics Statement

The studies involving human participants were reviewed and approved by Ethics Committee of Ile de France XI of Saint Germain en Laye (“Comité de protection des personnes, CPP,” reference number: 11040; 2011) and by the AFSSAPS (“Agence française de sécurité sanitaire et des produits de santé” reference number: B111102-90; 2011). The patients/participants provided their written informed consent to participate in this study.

## Author Contributions

OS-V, FP, M-LC, and J-ML: conceptualization and methodology. M-LC, OR, BB, PJ, VF, MM, JC, and YL: investigation and validation. JL: formal statistical analysis and visualization. LS: writing—original draft and visualization. OS-V, LS, FP, and J-ML: writing—review and editing. OS-V, FP, and J-ML: supervision. FP: funding acquisition. All authors contributed to the article and approved the submitted version.

## Funding

This work was funded by the Délégation à la recherche Clinique et à l'innovation, Centre Hospitalier de Versailles, France.

## Conflict of Interest

The authors declare that the research was conducted in the absence of any commercial or financial relationships that could be construed as a potential conflict of interest.

## Publisher's Note

All claims expressed in this article are solely those of the authors and do not necessarily represent those of their affiliated organizations, or those of the publisher, the editors and the reviewers. Any product that may be evaluated in this article, or claim that may be made by its manufacturer, is not guaranteed or endorsed by the publisher.
