# Supplementary material for: Platelet, Plasma, Urinary Tryptophan-Serotonin-Kynurenine Axis Markers in Hyperacute Brain Ischemia Patients: A Prospective Study
Source: Front Neurol. 2022 Jan 11;12:782317. doi: 10.3389/fneur.2021.782317 (PMC8787359; doi:10.3389/fneur.2021.782317)
Supplement: Supplementary file 1 [file Table_1.DOCX]

**SUPPLEMENTARY MATERIAL**

**SUPPLEMENTARY METHODS**

**1. Criteria for patient inclusion**

- age ≥ 18 years

- cerebral ischemia (transient ischemic attack, TIA without DWI changes on MRI, or acute ischemic stroke, AIS) with a time between the onset of symptoms, access to pre-hospital emergency care, and blood and urine sampling of <4.5 h.

- consent signed by the patient or a trusted person. In the event that the consent was signed by a trusted person, the signature of the consent was requested from the patient if his or her condition allowed it before discharge from the hospital or when the patient was seen again in consultation at the time of the three-month follow-up, if his or her condition allowed it and if he or she had not signed it on day 2 or before discharge from hospital.

**2. Criteria for patient** **exclusion**

- Patients with subarachnoid hemorrhage or cerebral hematoma

- Patients under guardianship, curatorship, or subject to legal protection

- Pregnancy

**3. Criteria for control inclusion**

- age ≥ 18 years

- signed consent

1. **Criteria for control exclusion**

- Pregnancy

- Under guardianship, curatorship, or subject to legal protection

- Diabetes

- Obesity (BMI >30)

- History of stroke or TIA

- Depression or melancholic state in progress, treated or untreated

- History of depression and suicide attempts

- Neuropsychiatric pathologies (bipolar disorder, depression, drug addiction, including known alcoholism)

- Treated or untreated migraine

- Participation in another therapeutic trial

- Progressive malignant disease (including carcinoid, treated or untreated)

- AIDS

- Recent or usual treatment with medications (such as some anti-depressant, anti-migraine, antiemetic, neuroleptic, and interferon drugs) that could interfere with the biological parameters being evaluated

- Treatment with anti-platelet or anticoagulation drugs

- Current hematological pathologies

- Known hemostasis disorders

- Known liver disease

**2. Details of clinical evaluation of patients**

The following elements were collected:

- Patient characteristics: sex, age, weight, and height

- Clinical history: hypercholesterolemia, smoking, arterial hypertension, diabetes, depression, current infection, and personal and family cardiovascular history

- Time of the signs of symptoms onset and management by emergency care

- The site of the cerebral infarction, if appropriate

On day 1 (D1), within 24 h from symptoms onset, AIS patients were assessed for depression in the two weeks prior to the stroke during a psychiatric examination using the Whooley Simplified Depression Scale and for impulsivity using the Baratt scale. If possible, this evaluation was carried out directly with the patient. Otherwise, it was carried out either with the patient's family and/or friends and/or with the patient's doctor.

1. **Anonymization**

Subjects were identified only by a "Research File Number", which consisted of a two-digit number followed by the letter P for patients and T for controls. Paired patients and controls had the same number. The Research File Number was assigned by the Scientific Research Coordinator upon confirmation of inclusion via the inclusion form. The list of matches between the identity of the subjects and their "Research File Number" was kept in a safe place by the principal investigator.

**SUPPLEMENTARY RESULTS**

1. **Study population – patients’ and controls’ demographic and clinical data**

**Supplementary Table I.** Recapitulation of patients’ demographic and clinical data. SRI: serotonin reuptake inhibitor.

| **Variable** | **N/median** | **% / Interquartile range** |
| --- | --- | --- |
| Age | 72 | [64.25 – 82.5] |
| Female sex | 14 | (50%) |
|  |  |  |
| Active smoking | 4 | (14%) |
| Treated arterial hypertension | 15 | (54%) |
| Atrial fibrillation | 3 | (11%) |
| Active infection | 0 | (0%) |
| Diabetes | 4 | (14%) |
| Arterial occlusion | 8 | (29%) |
| Treatment with SRI | 6 | (21%) |
| Anticoagulant/antithrombotic treatment | 25 | (89%) |
|  |  |  |
| Total cholesterol | 1.94 | [1.64 – 2.13] |
| Low-density lipoprotein | 1.28 | [0.95 – 1.4] |
| Triglycerides | 0.84 | [0.7 – 1.14] |
| Glucose | 6.55 | [5.4 – 7.6] |
| TIA | 4 | (14%) |
| Modified Rankin score | 2 | [0 – 3] |
| NIHSS score (Stroke patients) | 5 | [2 – 14] |
| ABCD2 score (TIA patients)  **Ischemic Cause** | 5 | [4 - 6] |
| Cardioembolic | 13 | (46%) |
| Undefined | 8 | (29%) |
| Neck vessel atheroma | 4 | (14%) |
| Aortic atheroma | 2 | (7%) |
| Lacunar stroke | 1 | (4%) |

**Supplementary Table II.** Recapitulation of controls’ demographic data.

| **Variable** | **N/median** | **% / Interquartile range** |
| --- | --- | --- |
| Age | 66.05 | [53.85 - 73.07] |
| Female sex | 12 | (41.4%) |
| Active smoking | 7 | (24.1%) |

1. **Supplementary data: recapitulation of global analysis (all patients vs all controls) at Day 0.**

**Supplementary Table III .** **Recapitulation of global analysis (all patients vs all controls) at Day 0.**

| **Variable** | **Median**  **(patients, n=28)** | **IQR**  **(patients, n=28)** | **Median**  **(controls, n=29)** | **IQR**  **(controls, n=29)** | **p-value** |
| --- | --- | --- | --- | --- | --- |
| Platelet 5-HT (nM) | 3 | [1.68 - 3.88] | 2.54 | [2.06 - 3.34] | 0.58 |
| Plasma 5-HT (nM) | 6.2 | [3.77 - 14.02] | 5.27 | [2.69 - 9.16] | 0.4 |
| Urinary 5-HT (nmol/mmol Creatinine) | 71.05 | [54.08 - 100.48] | 52.9 | [37.55 - 59.15] | 0.01 |
| Plasma 5-HIAA (nM) | 37 | [27.75 - 68.5] | 36 | [32 - 45] | 0.9 |
| Urinary 5-HIAA (umol/mmol Creatinine) | 3.8 | [2.9 - 5.6] | 2.09 | [1.7 - 2.86] | 0.003 |
| Platelet 5-HT_2A_R (fmol/mg protein) | 62.55 | [51.15 - 71.05] | 21.5 | [20.5 - 22.3] | 9.35 × 10^-8^ |
| Platelet SERT (pmol/mg protein) | 0.38 | [0.27 - 0.5] | 1.55 | [1.48 - 1.88] | 1.85 × 10^-5^ |
| MAO-A activity | 55.4 | [46.5 - 64.88] | 49 | [35.35 - 62.9] | 0.3 |
| Plasma K/T ratio | 5.56 | [4.72 - 6.67] | 3.74 | [2.86 - 4.52] | 1.03 × 10^-5^ |

1. **Supplementary data on Sex differences**

**Supplementary Table IV.** **Sex differences data analysis in patients at Day 0.** 5-HIAA, 5-Hydroxyindoleacetic acid; 5-HT, serotonin; IQR interquartile range; SERT, serotonin transporter.

| **Variable** | **Median**  **(Female, n=14)** | **IQR**  **(Female, n=14)** | **Median**  **(Male, n=14)** | **IQR**  **(Male, n=14)** | **p-value** |
| --- | --- | --- | --- | --- | --- |
| Platelet 5-HT (nM) | 3.50 | [1.5 - 3.9] | 2.80 | [2.2 - 3.8] | 0.79 |
| Plasma 5-HT (nM) | 4.85 | [3.52 - 10.1] | 9.10 | [3.9 - 15.75] | 0.3 |
| Urinary 5-HT (nmol/mmol Creatinine) | 73.10 | [50.27 - 121.8] | 71.05 | [57.88 - 99] | 0.9 |
| Plasma 5-HIAA (nM) | 40 | [22.5 - 106.5] | 34.00 | [28 - 49] | 0.5 |
| Urinary 5-HIAA (umol/mmol Creatinine) | 4.9 | [3.6 - 7.5] | 3.1 | [2.28 - 4.33] | 0.1 |
| Platelet 5-HT_2A_R (fmol/mg protein) | 67.9 | [57.62 - 82] | 55.55 | [50.25-62.6] | 0.07 |
| Platelet SERT (pmol/mg protein) | 0.37 | [0.28 - 0.42] | 0.4 | [0.29 - 0.94] | 0.4 |
| Urinary creatinine | 3.25 | [1.48 - 3.3] | 2.3 | [1.75 - 4.85] | 0.6 |
| MAO-A activity | 58 | [53.35 - 65.35] | 53.2 | [32.1 - 63.1] | 0.5 |
| Plasma K/T ratio | 5.7 | [4.68 - 6.69] | 5.38 | [5.07 - 5.86] | 1 |

1. **Supplementary data:** sub-analysis excluding TIA patients

**Supplementary Table V**. Sub-analysis at Day 0 excluding TIA patients.

| **Variable** | **Median**  **(patients, n=24)** | **IQR**  **(patients, n=24)** | **Median**  **(controls, n=29)** | **IQR**  **(controls, n=29)** | **p-value** |
| --- | --- | --- | --- | --- | --- |
| Platelet 5-HT (nM) | 3 | [1.68 - 3.88] | 2.54 | [2.06 - 3.34] | 0.6 |
| Plasma 5-HT (nM) | 6.2 | [3.9 - 14.02] | 5.27 | [2.69 - 9.16] | 0.41 |
| Urinary 5-HT (nmol/mmol Creatinine) | 65.45 | [50.5 - 97.62] | 52.9 | [37.55 - 59.15] | 0.03 |
| Plasma 5-HIAA (nM) | 35 | [27 - 53.5] | 36.00 | [32 - 45] | 0.66 |
| Urinary 5-HIAA (umol/mmol Creatinine) | 3.85 | [2.78 - 5.35] | 2.09 | [1.7 - 2.86] | 0.008 |
| Platelet 5-HT_2A_R (fmol/mg protein) | 62.55 | [51.15 - 68] | 21.5 | [20.5 - 22.3] | 2.1 × 10^-7^ |
| Platelet SERT (pmol/mg protein) | 0.4 | [0.27 - 0.5] | 1.55 | [1.48 - 1.89] | 9.92 × 10^-6^ |
| MAO-A activity | 56.7 | [52.67 - 65.82] | 49 | [35.35 - 62.9] | 0.09 |
| Plasma K/T ratio | 5.35 | [4.6 - 6.02] | 3.74 | [2.86 - 4.52] | 0.00012 |

1. **Supplementary data on evolution of TSK parameters over time**

**Supplementary Table V**I. E**volution of TSK parameters over time in patients.** The tables recapitulates values at the three time points and p-values of the paired comparisons. D0, day 0; D1, day 1; M3, three months.

| **Variable** | **D0 value (IQR range)** | **D1 value (IQR range)** | **M3 value (IQR range)** | **p-value D0-D1** | **p-value**  **D0-M3** | **p-value**  **D1-M3** |
| --- | --- | --- | --- | --- | --- | --- |
| Platelet 5-HT (nM) | 3 [1.68 - 3.88] | 3.2 [1.8 - 4.25] | 1.6 [0.35 - 2.65] | 0.379 | 0.937 | 0.855 |
| Plasma 5-HT (nM) | 6.2 [3.77 - 14.02] | 5.1 [3.1 - 10.5] | 4.5 [2.67 - 11.02] | 0.136 | 0.952 | 0.776 |
| Urinary 5-HT (nmol/mmol Creatinine) | 71.05 [54.08 - 100.48] | 32.8 [22.25 - 59.35] | 35.5 [31.5 - 55.7] | 0.016 | 0.007 | 0.461 |
| Plasma 5-HIAA (nM) | 37 [27.75 - 68.5] | 39[34-70] | 57 [38.5 - 75.25] | 0.740 | 0.397 | 0.209 |
| Urinary 5-HIAA (umol/mmol Creatinine) | 3.8 [2.9 - 5.6] | 2.3 [1.05 - 5.3] | 1.75 [1.2 - 4.2] | 0.272 | 0.224 | 0.554 |
| MAO-A activity | 55.4 [46.5 - 64.88] | 51.9 [29.87 - 130.88] | 47.75 [27.1 - 109.7] | 1 | 0.831 | 0.641 |
| Plasma K/T ratio | 5.56 [4.72 - 6.67] | 5.47 [4.36 - 6.34] | 5.74 [4.85 - 8.08] | 0.067 | 0.042 |  |

1. **Supplementary data on platelet aggregability**

**Supplementary Table VII.** Platelet aggregability before and after addition of serotonin (5-HT) in patients and controls (pooled).

| Variable | With ADP *0.5 µM* |  | With ADP *0.5 µM +* 5-HT 10 µM |  | P value |
| --- | --- | --- | --- | --- | --- |
| Aggregation Intensity | 33.01 [23.5 - 43] |  | 46.00 [34 - 55.38] |  | *P<10^-5^* |
| Aggregation Speed | 42.94 [27.15 - 56.07] |  | 57.00 [38.12 - 67.6] |  | *P<10^-5^* |

**Supplementary Table VIII**. Platelet aggregability in patients vs controls.

| Variable | Controls (ADP *0.5 µM +* 5-HT 10 µM) | Patients  (ADP *0.5 µM +* 5-HT 10 µM) | P value |
| --- | --- | --- | --- |
| Aggregation Intensity | 35.5 [25.88 - 53.88] | 47.75 [45.13 - 57.02] | 0.0769 |
| Aggregation Speed | 49.5 [32.2 - 67.85] | 59.38 51.34 - 66.25] | 0.0927 |

**SUPPLEMENTARY FIGURES**

**Supplementary Figure I. Study design**. AIS, acute ischemic stroke; TIA, transient ischemic attack; TSK, tryptophan-serotonin-kynurenine

**Supplementary Figure II. Evolution of plasma kynurenine/tryptophan ratio over time in patients.** Asterisks indicate P values <0.05; D0, day 0; D1, day 1; M3, three months.

**Supplementary Figure III. Evolution of plasma and urinary markers over time.** A, Plasma serotonin (5-HT). B Plasma 5-HIAA (5-hydroxyindole acetic acid). C, Urinary 5-HT. D, Urinary 5-HIAA. Asterisks indicate P values <0.05; D0, day 0; D1, day 1; M3, three months.
